# Agreement between parent-report and EMR height, weight, and BMI among rural children

**DOI:** 10.3389/fnut.2024.1279931

**Published:** 2024-03-01

**Authors:** Benjamin A. Potts, G. Craig Wood, Lisa Bailey-Davis

**Affiliations:** Center for Obesity and Metabolic Research, Geisinger Health System, Danville, PA, United States

**Keywords:** remote anthropometry, parent-report anthropometry, child weight classification, child growth monitoring, pediatric telemedicine, BMI corrections, obesity detection, healthcare during COVID-19

## Abstract

**Introduction:**

Remote anthropometric surveillance has emerged as a strategy to accommodate lapses in growth monitoring for pediatricians during coronavirus disease 2019 (COVID-19). The purpose of this investigation was to validate parent-reported anthropometry and inform acceptable remote measurement practices among rural, preschool-aged children.

**Methods:**

Parent-reported height, weight, body mass index (BMI), BMI z-score, and BMI percentile for their child were collected through surveys with the assessment of their source of home measure. Objective measures were collected by clinic staff at the child’s well-child visit (WCV). Agreement was assessed using correlations, alongside an exploration of the time gap (TG) between parent-report and WCV to moderate agreement. Using parent- and objectively reported BMI z-scores, weight classification agreement was evaluated. Correction equations were applied to parent-reported anthropometrics.

**Results:**

A total of 55 subjects were included in this study. Significant differences were observed between parent- and objectively reported weight in the overall group (−0.24 kg; *p* = 0.05), as well as height (−1.8 cm; *p* = 0.01) and BMI (0.4 kg/m^2^; *p* = 0.02) in the ≤7d TG + Direct group. Parental reporting of child anthropometry ≤7d from their WCV with direct measurements yielded the strongest correlations [*r* = 0.99 (weight), *r* = 0.95 (height), *r* = 0.82 (BMI), *r* = 0.71 (BMIz), and *r* = 0.68 (BMI percentile)] and greatest classification agreement among all metrics [91.67% (weight), 54.17% (height), 83.33% (BMI), 91.67% (BMIz), and 33.33% (BMI percentile)]. Corrections did not remarkably improve correlations.

**Discussion:**

Remote pediatric anthropometry is a valid supplement for clinical assessment, conditional on direct measurement within 7 days. In rural populations where socioenvironmental barriers exist to care and surveillance, we highlight the utility of telemedicine for providers and researchers.

## 1 Introduction

Childhood obesity poses an imminent threat to the wellbeing of children worldwide (1, 2). Obesity is associated with non-communicable diseases such as cardiovascular disease (CVD), type 2 diabetes mellitus (T2DM), musculoskeletal disorders, cancer, and increased mortality risk (2).

The coronavirus disease 2019 (COVID-19) pandemic has placed increased strain on children at risk or already classified as overweight or obese. The prevalence of childhood obesity has increased rapidly from 19.3 to 22.4% amid the pandemic (3). Children’s ability to achieve adequate physical activity has been hampered and supplanted with increased time spent engaged in sedentary behavior (4). The metabolic storm generated from the upheaval of physical activity exacerbates the progression of the disease state and worsens obesity-related sequelae (5).

A constellation of factors influences the presence of childhood obesity; however, the living environment and geography may predispose children to a greater likelihood of developing overweight or obesity. Specifically, children in rural areas face higher odds of developing obesity than those in urban areas (6–8). An array of socioenvironmental factors is associated with obesogenic disparities observed in rural children, some of which function as barriers to clinical care (8, 9). Rural patients face long clinic commutes, and those from low-income households may have unreliable transportation. The American Academy of Pediatrics (AAP) identifies telemedicine as an essential strategy to reduce such barriers (10). While utilization has increased during the pandemic, broadband technology access presents a novel barrier for rural patients (11–13).

Telemedicine offers convenience but presents challenges for physical exams, as indicated in monitoring child growth. The use of age- and sex-specific body mass index (BMI) as an acceptable metric for classifying childhood overweight or obesity by providers was established by the US Preventative Services Task Force (USPSTF) in 2005 (14). The gold standard for anthropometric surveillance data is objectively measured height and weight, used to calculate BMI and related BMI indices (15). BMI indices are plotted on age- and sex-specific growth charts generated from population samples, establishing reference norms and allowing providers to determine where a child falls along the reference continuum (15). While variable for children in normal weight ranges due to variance in lean mass, BMI is associated with acceptable sensitivity as an indicator of adverse weight in children with an excess of adiposity (16).

Parent-proxy anthropometric reporting is an emerging strategy to supplement well-child visit (WCV) delays and demonstrate pediatric telemedicine utility. However, there are challenges in obtaining these metrics annually (15). WCV delays during the pandemic resulted in an aggravation of the existing concerns regarding annual anthropometric surveillance (17). To combat these concerns, a shift toward self- or parent-proxy-reported height and weight has been initiated to supplement clinical monitoring (15, 18–28). Measurement continuity is crucial when seeking to collect valid proxy-report measures. Smart scales or other technologically advanced tools may be optimal, but socioeconomically disadvantaged populations (i.e., rural) rarely have sufficient access to technology allowing them to utilize these instruments (23). Studies using widely available measurement tools display utility, though they may be subject to the risk of reporting bias (23). Further, home measurements are more acceptable when solely using direct measurements (19). Bias mitigation strategies should include consideration of the time gap (TG) between parent-proxy reported and WCV anthropometrics (23, 29–31). Parental underreporting of child weight and/or overreporting of child height are common in studies assessing the relationship between self-report and objective measures (18, 21, 22, 26). These inaccuracies are pronounced to a greater degree in children with pre-existing overweight or obesity (22, 32). Chronic misreporting can lead to chronic misrepresentation of BMI accuracy and the identification of overweight and obesity.

Correction equations for parent-reported height and weight have been assessed for their ability to ameliorate parental reporting bias (29–31, 33–39). These equations are derived from individual datasets, rendering them unique to sociodemographic characteristics from their reference sample (29, 35, 37–39). As pediatric healthcare delivery in rural settings faces residual scheduling challenges and loss of follow-up amid a transition to telemedicine, validation of parent-reported height and weight in underrepresented age groups is pivotal (17, 40, 41). To date, there is limited evidence utilizing correction equations in preschool-aged children. Hence, the objectives of the present investigation are: (1) determining correlations between parent-reported and objective clinical measures (weight, height, BMI, BMI z-score, and BMI percentile); (2) assessing the impact of the TG between parent and clinical report on these relationships; (3) discerning whether the source of the home measure impacts the relationships (e.g., measured vs. estimated values); and (4) evaluating the utility of corrections and their ability to improve correlations between parent-reported and objective clinical measures.

## 2 Methods

### 2.1 Study context—secondary analysis using data from the ENCIRCLE study

The patient-cliNic-Community Integration to prevent obesity in Rural preschool ChiLdrEn (ENCIRCLE) study is a pragmatic, cluster randomized controlled trial (RCT) that was conducted across Geisinger primary care clinics in central and northeast Pennsylvania (42). This study was designed to compare the effectiveness of clinic, patient-clinic, or patient-clinic-community interventions to attenuate the prevalence of obesity among preschool-aged children exposed to obesogenic environments (42). Many of these clinic locations are rural and serve an array of socioeconomic backgrounds within their respective communities.

Primary care providers (PCPs) from family medicine (*n* = 51) or pediatric (*n* = 54) clinics were randomized to one of the three potential intervention arms: standard WCV, patient-reported outcome (PRO) enhanced WCV, or PRO enhanced WCV plus Food Care. The WCV arm consisted of routine care aligned with clinical practice guidelines, including BMI screening and provider-led counseling (43). The PRO WCV and PRO WCV plus Food Care arms both integrate the parent-reported Family Nutrition and Physical Activity (FNPA) risk assessment tool into the WCV (44, 45). Food Care enhancements to the intervention involved patient referrals to community health professionals responsible for providing evidence-based obesity prevention in conjunction with telehealth guidance on economical dietary planning (46). The ENCIRCLE study was incidentally launched in March 2020, in concordance with the COVID-19 pandemic. Telehealth has emerged as a critical component in maintaining continuous healthcare delivery amid efforts to mitigate transmission during the pandemic. The ENCIRCLE study was approved by Geisinger’s Institutional Review Board and is presently registered with ClinicalTrials.gov (NCT04406441).

### 2.2 Study participants

This study incorporates a subset of the ENCIRCLE study population that has both clinical and parent-reported height and weight metrics (*n* = 55) for children.

### 2.3 Data collection

#### 2.3.1 Parent-proxy reported anthropometrics

Data collection from consenting parents included self-reported children’s age, biological sex, race/ethnicity, children’s height and weight, relationship to the child, educational level, annual household income indices, and employment status. Respondents indicated whether weight and height were estimated or measured directly at home. Values were reported to the nearest inch/cm and pound/kilogram. A screenshot of these questions is included (Supplementary Figure S1) as the parent would view them. No details about home equipment were collected.

#### 2.3.2 Objectively assessed anthropometrics

Children’s height and weight were recorded using standardized procedures during WCVs by trained clinic staff. Height was measured to the nearest 0.1 cm using a stadiometer (SECA 264), and weight was measured to the nearest 0.1 kg using a calibrated digital scale (Health-o-meter 599KL).

#### 2.3.3 Calculation of BMI, BMI z-score, and BMI percentile

Parent-proxy and objectively measured BMI were calculated from parent-reported and objectively measured height and weight, respectively. Children’s BMI z-score and BMI percentile for age and sex were calculated from the Centers for Disease Control (CDC) programs designed for children from 2 to 19 years of age.

#### 2.3.4 Weight classifications

Sex-specific BMI-for-age percentiles were calculated in the EHR system to identify children by weight status: normal weight (> 5th and < 85th), overweight (≥ 85th and < 95th), obese (≥ 95th and < 99th), and severely obese (≥ 99th).

#### 2.3.5 Population sociodemographic characteristics

Demographic characteristics of the ENCIRCLE study population were recorded into the REDCap database during the eligibility screening process and inclusion/exclusion survey criteria from consenting parents. Additional characteristics were collected during a study team follow-up call.

#### 2.3.6 Correction methods

Correction equations were derived using an univariate analysis to determine subject characteristics to be applied to the correction model. Adjusted R^2^ statistics assess the fit of the model. The model in the present investigation was extrapolated from parents reporting their child’s height and weight ≤ 7 days prior to the WCV (*n* = 37) to accommodate the influence of the TG between parent-proxy and objective measurements. Indirect corrections for parent-proxy reported BMI, i.e., applying correction equations to parent-proxy reported height and weight to subsequently calculate a corrected BMI, were employed. Correction models were uniquely generated for our given dataset due to the lack of established correction models for preschool-aged children and consideration of previous literature that has advised against the application of correction equations to multiple datasets (34, 47, 48). Correction modeling procedures were carried out in SAS (SAS Institute, Cary, NC, USA).

### 2.4 Statistical analyses

To mitigate potential reporting bias in parent-proxy measurements, only anthropometrics that were reported prior to the child’s WCV were included in analyses. Sociodemographic factors were included to characterize the study population. Differences between parent-proxy and objective clinic measurements were reported to describe the distribution of height, weight, BMI, BMI z-score, and BMI percentile. The calculation of age- and sex-specific BMI z-scores and percentiles was performed using SAS procedures provided by the CDC. A stratification based on the TG was performed to identify differences between parent-proxy and objective anthropometrics. TG differences were applied to compare measurements that occurred ≤7 days before the WCV against measurements that occurred >7 days before the WCV. REDCap surveys were given to participating parents, representing each parent–child dyad. Parents were asked whether their reported measurements (height or weight) were directly measured or estimated. To differentiate subjects whose heights and weights were directly measured, TG groups (≤7 days and > 7 days) were subject to additional stratification based on survey responses.

Independent samples *t*-tests were employed to assess between-group differences for child demographic characteristics. Paired samples *t*-tests assess differences between parent-proxy and objectively reported measures (height, weight, BMI, BMI z-score, and BMI percentile). The significance level was set at a value of *p* of ≤0.05. Pearson’s correlation coefficient (R) acts as the ratio of covariance between variables and was calculated to assess the relationship between parent-proxy and objective measures. Scatter plots comparing parent-proxy and objective measures were generated to display correlations and the data distribution.

Bland–Altman plots were used to assess agreement between parent-proxy and objective measurements for height, weight, BMI, BMI z-score, and BMI percentile (47).

Sensitivity (true positive) and specificity (true negative) analyses were employed in the present analysis to discern weight classification error and agreement based on parent-proxy BMI z-score. In the present study, sensitivity identified the proportion of children who are objectively classified as obese and concurrently classified as obese by parent-proxy reports. Specificity identified the proportion of children who are objectively classified as non-obese and concurrently classified as non-obese by parent-proxy reports. Cutoff values for BMI z-score weight categories are described as normal (−2 SD > BMI > 1 SD), overweight (1 SD > BMI > 2 SD), and obese (BMI > 2 SD) in accordance with previous literature (48). Positive predictive value (PPV), negative predictive value (NPV), and accuracy were also calculated in the present analysis (48).

Cohen’s Kappa quantified the agreement of weight categorization (normal weight, overweight, or obese) between parent-proxy and objective measurements using calculated BMI z-scores. Cohen’s Kappa is interpreted by a range of scores from 0.0 to 1.0; <0 indicating a ‘less than chance’ agreement, 0.01–0.20 indicating a ‘slight’ agreement, 0.21–0.40 indicating ‘fair’ agreement, 0.41–0.60 indicating ‘moderate’ agreement, 0.61–0.80 indicating ‘substantial’ agreement, and 0.81–0.99 indicating ‘almost perfect’ agreement (49). The intraclass correlation coefficient (ICC) assessed the outcome variation between parent-proxy and objective measures (50). ICC is interpreted along a spectrum from 0.0 to 1.0, where values <0.5 are indicative of ‘poor reliability,’ values between 0.5 and 0.75 are indicative of ‘moderate’ reliability, values between 0.75 and 0.9 are indicative of ‘good’ reliability, and values >0.9 indicate ‘excellent’ reliability (51). Histograms were created, displaying the frequency of agreement between parent-proxy and objective measures.

## 3 Results

A total of 55 parent–child dyads represent the overall study population. Most children in this study were female (53%). The mean age at the time of parental reporting was 46.4 months, and 46.9 months at their WCV. The average TG between parent-report and WCV was 16.9 days. Demographic characteristics for all stratification groups are described in Table 1. Certain parental demographic data (i.e., parent gender, ethnicity, race, education, or employment) were calculated based on a smaller reference sample due to the presence of occasional missing survey report data.

**Table 1:** Demographic characteristics of the study population.

Participant (Child) Characteristics⁎	Overall (*n* = 55)	≤7d TG (*n* = 37)	>7d TG (*n* = 18)	≤7d TG + Direct (*n* = 24)	>7d TG + Direct (*n* = 15)
Child age (mo)^†^					
Age at report	46.4 (11.0)	47.0 (11.4)	45.1 (10.0)	47.4 (10.8)	45.8 (10.4)
Age at WCV	46.9 (11.0)	47.4 (11.4)	46.6 (10.0)	47.4 (10.8)	47.2 (10.5)
Time gap (d)^a†^	16.9 (25.3)	2.7 (2.0)	45.3 (26.4)	2.3 (2.1)	42.3 (26.3)
Child sex^†^	***n* = 55**	***n* = 37**	***n* = 18**	***n* = 24**	***n* = 15**
Male	26 (47)	16 (43)	10 (56)	11 (46)	8 (53)
Female	29 (53)	21 (57)	8 (44)	13 (54)	7 (47)
Parent characteristics^⁑^					
Sex^‡^	***n* = 53**	***n* = 35**	***n* = 18**	***n* = 23**	***n* = 15**
Male	1 (2)	1 (3)	0 (0)	1 (4)	0 (0)
Female	52 (98)	34 (97)	18 (100)	22 (96)	15 (100)
Ethnicity^‡^	***n* = 54**	***n* = 36**	***n* = 18**	***n* = 23**	***n* = 15**
Hispanic	2 (4)	1 (3)	1 (6)	0 (0)	1 (7)
Non-Hispanic	52 (96)	35 (97)	17 (94)	23 (100)	14 (93)
Race^‡^	***n* = 54**	***n* = 36**	***n* = 18**	***n* = 23**	***n* = 15**
Caucasian	52 (96)	35 (97)	17 (94)	22 (96)	14 (93)
African American	1 (2)	0 (0)	1 (6)	0 (0)	1 (7)
Mixed race	1 (2)	1 (3)	0 (0)	1 (4)	0 (0)
Education^‡^	***n* = 54**	***n* = 36**	***n* = 18**	***n* = 23**	***n* = 15**
HS/GED	21 (39)	12 (33)	9 (50)	8 (35)	8 (53)
College degree	15 (28)	10 (28)	5 (28)	6 (26)	3 (20)
Graduate degree	14 (26)	10 (28)	4 (22)	7 (30)	4 (27)
Other^b^	4 (7)	4 (11)	0 (0)	2 (9)	0 (0)
Employment^‡^	***n* = 45**	***n* = 30**	***n* = 15**	***n* = 20**	***n* = 13**
Full-time	19 (42)	14 (47)	5 (33)	7 (35)	3 (23)
Part-time	14 (31)	9 (30)	5 (33)	8 (40)	5 (38)
Unemployed	8 (18)	5 (17)	3 (20)	3 (15)	3 (23)
Other^c^	4 (9)	2 (7)	2 (13)	2 (10)	2 (15)

^⁎^Independent sample *T*-test was utilized to compare differences in child characteristics within each stratification group; no significant differences were detected.

^†^Values are presented as Mean ± Standard Deviation (SD).

^a^Time gap refers to the number of days (d) between the time of parent-reporting and well-child visit (WCV) assessment.

^⁑^Sum of parent characteristic variables differs due to missing data.

^‡^Values are presented as n (n%).

^b^Other educational backgrounds reported include trade school, some college, or a doctorate.

^c^Other employment situations were unspecified.

Regardless of the TG, parents tend to report their child’s height (74.5%) and weight (85.5%) as “1” (direct) most of the time. Parents reporting their child’s measurements >7 days before their WCV utilize direct measurements more for height (83.3%) and weight (94.4%) than those reporting ≤7 d (70.3, 81.1%, respectively).

Table 2 describes means, differences, and agreement analyses (ICC, Pearson’s R) for parent- and objectively reported anthropometrics for the overall study population, and individual stratification groups. A significant difference between parent-reported weight and objectively measured weight was detected across the overall sample (*p* = 0.05). Significant differences were detected for height (*p* = 0.01) and BMI (*p* = 0.02) in the ≤7d TG + Direct group.

**Table 2:** Means, differences, and agreement between parent-proxy reported measures and objective measures from clinic visits.

	Parent-report^a^	Objective report^a^	Mean difference^b†^	Agreement	
				ICC^c^	Pearson’s R^d^
Overall group (*n* = 55)					
Weight (kg)	17.1 (3.4)	17.4 (3.5)	−0.2 (0.9)^‡^	0.97	0.97
Height (cm)	101.3 (11.4)	102.2 (9.5)	−0.8 (4.8)	0.89	0.91
BMI (kg/m^2^)	16.7 (1.8)	16.5 (1.6)	0.1 (1.6)	0.57	0.57
BMI z-score	0.6 (1.2)	0.6 (1.1)	0.1 (1.1)	0.55	0.55
BMI %	65.6 (30.5)	66.9 (27.0)	−1.2 (28.9)	0.50	0.50
≤7d TG group (*n* = 37)					
Weight (kg)	17.5 (3.6)	17.7 (3.6)	−0.2 (0.6)	0.98	0.98
Height (cm)	101.6 (12.5)	102.7 (9.6)	−1.1 (4.7)	0.91	0.94
BMI (kg/m^2^)	16.9 (1.8)	16.7 (1.6)	0.3 (1.5)	0.61	0.62
BMI z-score	0.8 (1.2)	0.7 (1.0)	0.2 (1.0)	0.58	0.59
BMI %	70.5 (29.8)	68.4 (26.1)	2.1 (26.9)	0.53	0.53
>7d TG group (*n* = 18)					
Weight (kg)	16.5 (3.2)	16.8 (3.5)	−0.3 (1.2)	0.93	0.94
Height (cm)	100.7 (9.1)	101.0 (9.4)	−0.3 (5.0)	0.86	0.85
BMI (kg/m^2^)	16.2 (1.7)	16.3 (1.5)	−0.2 (1.7)	0.44	0.43
BMI z-score	0.2 (1.2)	0.4 (1.3)	−0.2 (1.2)	0.48	0.47
BMI %	55.6 (30.4)	63.7 (29.1)	−8.2 (31.5)	0.44	0.44
≤7d TG + Direct group (*n* = 24)					
Weight (kg)	17.2 (3.0)	17.4 (2.9)	−0.2 (0.4)	0.99	0.99
Height (cm)	101.2 (9.6)	103.0 (8.6)	−1.8 (2.9)^‡^	0.93	0.95
BMI (kg/m^2^)	16.8 (1.5)	16.4 (1.4)	0.4 (0.9)^‡^	0.79	0.82
BMI z-score	0.8 (0.9)	0.5 (1.0)	0.3 (0.7)	0.67	0.71
BMI %	72.2 (25.7)	64.8 (26.6)	7.3 (20.8)	0.69	0.68
>7d TG + Direct group (*n* = 15)					
Weight (kg)	16.4 (3.4)	16.6 (3.6)	−0.2 (1.3)	0.93	0.93
Height (cm)	99.2 (7.9)	100.3 (9.9)	−1.1 (4.5)	0.87	0.89
BMI (kg/m^2^)	16.5 (1.6)	16.3 (1.6)	0.2 (1.6)	0.54	0.53
BMI z-score	0.5 (1.1)	0.3 (1.4)	0.1 (1.0)	0.66	0.67
BMI %	62.6 (27.2)	62.4 (31.2)	0.1 (24.4)	0.67	0.66

^†^Paired samples *T*-test; ^‡^significant difference (Overall; Weight [*p* = 0.05], ≤7d TG + Direct; Height [*p* = 0.01], ≤7d TG + Direct; BMI [*p* = 0.02]).

^a^Values shown as mean ± standard deviation (SD).

^b^Mean difference calculated by subtracting the mean of the objectively reported values from the mean of the self-reported values (Mean_Self_-Mean_Obj_).

^c^Intraclass correlation coefficient (ICC); <0.50 indicates poor reliability, 0.50–0.75 indicates moderate reliability, 0.75–0.90 indicates good reliability, and > 0.90 indicates excellent reliability.

^d^Pearson’s correlation coefficient (Pearson’s R); values are interpreted on a continuum between − 1 (perfect negative correlation), 0 (no correlation) and + 1 (perfect positive correlation).

Weight classification agreement measured using Cohen’s Kappa for BMI z-score within the overall population yielded a Kappa coefficient of 0.22, indicating fair agreement.

Sensitivity, specificity, PPV, NPV, and accuracy are calculated using parent-proxy reported BMI z-score for weight classification, derived from parent-reported height and weight (Table 3). Sensitivity in normal weight subjects decreased as stratification level increased across the three groups (66, 60, 53%), and specificity increased similarly across the three groups (55, 83, 86%). Overweight specificity decreased across the three groups (67, 60, and 55%), and sensitivity increased (38, 71, and 100%). Sensitivity and specificity did not notably change among obese subjects.

**Table 3:** Sensitivity, specificity, positive predictive value, negative predictive value, and accuracy of parent-reports for predicting weight classification with parent-reported BMI z-score.

	Parent-report overall (*n* = 55)			Parent-report ≤ 7d TG (*n* = 37)			Parent-report ≤ 7d TG + Direct (*n* = 24)		
	NM (n%)	OW (n%)	OB (n%)	NM (n%)	OW (n%)	OB (n%)	NM (n%)	OW (n%)	OB (n%)
Sensitivity^a^	66	38	60	60	71	50	53	100	50
Specificity^b^	55	67	98	83	60	97	86	55	100
PPV^c^	72	26	75	88	29	67	90	31	100
NPV^d^	48	78	96	50	90	94	43	100	96
Accuracy^e^	62	60	95	68	62	92	63	63	96

NM, normal weight; OW, overweight; OB, obese.

^a^Sensitivity = True Positives / (True Positives + False Negatives).

^b^Specificity = True Negatives / (True Negatives + False Positives).

^c^Positive Predictive Value = True Positives / (True Positives + False Positives).

^d^Negative Predictive Value = True Negatives / (True Negatives + False Negatives).

^e^Accuracy = (True Positives + True Negatives) / Total “n.”

Table 4 describes the classification accuracy of parent-reported anthropometrics for all stratification groups. Unanimously, the ≤7d TG + Direct group displayed the greatest level of classification agreement (weight, 91.67%; height, 54.17%; BMI, 83.33%; BMI z-score, 91.67%; and BMI percentile, 33.33%). Conversely, the >7d TG + Direct group displayed the lowest rates of classification agreement for height (26.67%) and weight (60%), while the >7d TG group displayed the lowest rates of classification agreement for all three BMI indices (BMI, 38.89%; BMI z-score, 55.56%; BMI percentile, 22.22%). An agreement gradient emerged across classification groups, ranking highest to lowest agreement from ≤7d TG + Direct, ≤7d TG, Overall, >7d TG + Direct, and > 7d TG.

**Table 4:** Accuracy of parent-proxy reported anthropometric data for weight, height, BMI, BMI z-score, and BMI percentile.

Accuracy of parent-report data					
Classification^†^	Overall (*n* = 55)	≤7d TG (*n* = 37)	>7d TG (*n* = 18)	≤7d TG + Direct (*n* = 24)	>7d TG + Direct (*n* = 15)
Weight (kg)^a^	n%				
Accurate	67.27	81.08	66.67	91.67	60.00
Underestimation	27.27	16.22	16.67	8.33	20.00
Overestimation	5.45	2.70	16.67	0.00	20.00
Height (cm)^b^	n%				
Accurate	36.36	40.54	27.78	54.17	26.67
Underestimation	43.64	43.24	44.44	41.67	53.33
Overestimation	20.00	16.22	27.78	4.17	20.00
Body mass index (BMI)^c^	n%				
Accurate	58.18	67.57	38.89	83.33	53.33
Underestimation	18.18	10.81	33.33	16.67	33.33
Overestimation	23.64	21.62	27.78	0.00	13.33
BMI z-score^d^	n%				
Accurate	72.73	83.78	55.56	91.67	73.33
Underestimation	10.91	5.41	22.22	8.33	13.33
Overestimation	16.36	10.81	22.22	0.00	13.33
BMI percentile^e^	n%				
Accurate	27.27	29.73	22.22	33.33	26.67
Underestimation	32.73	27.03	44.44	20.83	33.33
Overestimation	40.00	43.24	33.33	45.83	40.00

^†^All accuracy, underestimations, and overestimations reference the comparison of parent-reported measurements to well-child visit (WCV) measurements as the standard.

^a^Accurate within ± 1 kg, underestimation by > 1 kg, overestimation by > 1 kg.

^b^Accurate within ± 2 cm, underestimation by > 2 cm, overestimation by > 2 cm.

^c^Accurate within ± 1 BMI pt, underestimation by > 1 BMI pt, overestimation by > 1 BMI pt.

^d^Accurate within ± 1 SD, underestimation by > 1 SD, overestimation by > 1 SD.

^e^Accurate within ± 5%, underestimation by > 5%, overestimation by > 5%.

The limits of agreement (LOA) are representative of the mean difference ± 1.96 standard deviations and are calculated and displayed within each respective Bland–Altman plot (Supplementary Figures S2A–E). These include LOA for weight (1.48, −1.95), height (8.65, −10.31), BMI (3.2, −2.95), BMI z-score (2.19, −2.08), and BMI percentile (55.39, −57.87).

Correction models were applied to parent-reported height and weight, as subsequently described.

- Corrected Weight = 0.489 + (0.984 * Weight_Self_)
- Corrected Height = 38.952 + (0.462 * Height_Self_) + (0.350 * months)

Correlations (Pearson’s R) were calculated to assess the agreement between corrected parent- and objectively reported height, weight, and indirectly calculated BMI. In the ≤7d TG + Direct group (*n* = 37), the weight correlation remained unchanged (0.99), decreased for height (0.95 to 0.94), and decreased for indirect BMI (0.82 to 0.72). In the >7d TG + Direct group (*n* = 15), weight correlation remained unchanged (0.93), height remained unchanged (0.89), and indirect BMI improved (0.53 to 0.63).

## 4 Discussion

Amid strong agreement between parent- and objectively reported height and weight within the overall population, BMI metrics (BMI, BMI z-score, and BMI percentile) were poorly correlated. Thus, we sought to explore stratification methods and corrections to strengthen this agreement. We observed the marked influence of the TG, noting that measurements recorded and reported within 7 days displayed higher indications of agreement when compared to those greater than 7 days from the child’s WCV. These improvements were augmented by controlling for the source of the home measure (i.e., including only direct measures). However, our application of corrections to parent-reported height, weight, and BMI did not accentuate agreement. Our findings support the utility of remote anthropometry under the conditions of reporting within 7 days and confirming that the child’s at-home measurement was direct.

Objective assessments performed by trained clinical staff are considered the gold standard of anthropometric assessment (15, 52–55). As telemedicine becomes a staple of clinical practice in the wake of COVID-19, the validation of remote anthropometry in children has garnered increased attention (15, 18–28). Previous research has leveraged pre-recorded at-home video instruction (23), live at-home video conference (37), and smart-scale technology (56, 57) when collecting height and weight remotely. In the present study, we neither provided home equipment nor collected specific information regarding tools that parents used to measure their children on their own accord. Regardless, we found that direct measurements indicated by survey responses positively impacted the agreement between parental and clinical raters. Skinner et al. underscore the importance of clarifying the source of the home measure, finding that younger children were more likely to be misclassified into an incorrect weight classification following parental guessing (19). Our findings are further supported by a recent investigation by Forseth et al. where negligible differences were found between the use of at-home, study-provided, or objectively measured (school-based stadiometer) height and weight among rural children (23). While measurement instructions may provide a feasible strategy to mitigate reporting bias, broadly accessible tools for home-based measurements are acceptable.

Our study is among the few to identify the TG between reported measures and objective assessments as a critical component of moderating agreement (29, 58). Cheng et al. provide a framework for examining the TG as it relates to reporting accuracy and stratifying their patient population into those reporting within 7 and 30 days, respectively. In line with our findings, this group found that reporting within 7 days of objective measurement was associated with a lesser difference between reported and objective assessments. We found that controlling for TGs within 7 days improved agreement for the overall sample. The magnitude of agreement was amplified when additional control for the source of the home measure was included. Our validation provides clinicians and researchers with an opportunity to enhance the accuracy of their use of remote anthropometry in telemedicine.

Accurate weight classification is critical for gauging the breadth of childhood overweight and obesity. The WHO recommends using BMI z-scores in research for the sake of continuity (59). Using a clinically measured BMI z-score as an anchor, we found parental reporting accuracy to gradually improve when controlling for the TG within 7 days, followed by a compounded increase in accuracy when controlling for the source of the home measure. Several reviews have identified a high prevalence of parental weight misclassification for their children (60–64). A review by Sherry et al. found parental reporting of BMI to be 55–76% sensitive for identification, and the prevalence of overweight decreased by −0.4 to −17.7% when calculated BMI was derived from parental reports, indicating chronic underreporting (65). These findings align with ours, where overweight sensitivity was low for the overall parent-reported sample (38%), indicating poor ability to correctly classify children as overweight. Only when additional control was integrated for the TG within 7 days, a direct source of home measure, did sensitivity improve (38, 71, and 100%, respectively). While limited, the literature suggests that the rationale for parental reporting bias is potentially due to factors such as digit preference, inconsistent assessment timepoint, rounding error, and social desirability bias that may create misconceptions about their children’s weight (32, 66–68). Further elucidation of socioenvironmental and interpersonal influences on parental weight misclassification is warranted.

Under- or overreporting of child height and weight typically yields poor agreement between BMI calculated from parental reporting and BMI calculated from objective measures (26, 29). Underestimations of BMI have been reported to the degree of 0.5 kg/m^2^ (26, 37) and 0.6 kg/m^2^, respectively (69). Shields et al. have identified BMI overreporting as well, by a margin of 0.7 kg/m^2^ based on parental reports (34). Our study finds BMI to be overestimated by 0.13 kg/m^2^, despite underreporting of height and weight within the overall sample. Correction modeling provides an opportunity to combat misreporting and improve agreement between parent- and objectively reported height, weight, and BMI (29–31, 33–39). In one study utilizing correction modeling, agreement quantified by ICC improved from 0.33 to 0.64 for the classification of overweight or obese status after indirect corrections were applied for reporting within 7 days (29). Ghosh-Dastidar et al. show increased sensitivity for obesity regardless of sex and smaller RMSE values using indirect corrections, while noting heterogeneity of model applicability due to gender and outcome (37). We sought to control out-of-context equation applications by generating our own equations for our population. We align our use of ‘indirect’ BMI corrections with research that has shown favorable outcomes using this modeling technique (29, 37). However, no improvements in agreement parameters were found in our study. We speculate that the heightened levels of agreement we were able to achieve when controlling for the TG and source of home measure negated the capacity for corrections to further improve these relationships.

We provide validation for the concordance between parent-reported and objectively measured data, although the present study is not without limitations. Our omission of collecting information about home equipment used to measure children’s height and weight stymies the evaluation of factors that may contribute to parental reporting bias. Additionally, our predominantly low-income, rural population may be inordinately impacted by a lack of internet access, hindering their capacity to engage with telehealth (70, 71). Additional limitations include the sample size and geographical constraints that restrict the generalizability of these findings to populations with comparable demographic characteristics. Overall, the present study serves to inform emergent literature regarding the use of parent-reported anthropometrics during the pandemic. These outcomes will advise clinicians, healthcare administrators, policymakers, and researchers who seek to leverage remote anthropometry as a supplement for clinical measures.

## Data availability statement

The data analyzed in this study is subject to the following licenses/restrictions: the data from the present study are available from the corresponding author upon request. Requests to access these datasets should be directed to LB-D, ldbaileydavis@geisinger.edu.

## Ethics statement

The studies involving humans were approved by the Declaration of Helsinki, and ethical approval was obtained from the Institutional Review Board of Geisinger (#2020–0207, Version 1.19, 9/7/2022). Informed consent and parental permission were obtained from all participants. The studies were conducted in accordance with the local legislation and institutional requirements. Written informed consent for participation in this study was provided by the participants’ legal guardians/next of kin.

## Author contributions

BP: Formal analysis, Investigation, Methodology, Validation, Writing – original draft, Writing – review & editing. GW: Data curation, Formal analysis, Software, Supervision, Writing – review & editing. LB-D: Conceptualization, Funding acquisition, Methodology, Project administration, Resources, Supervision, Visualization, Writing – review & editing.
