# Supplementary material for: Agreement between parent-report and EMR height, weight, and BMI among rural children
Source: Front Nutr. 2024 Mar 1;11:1279931. doi: 10.3389/fnut.2024.1279931 (PMC10940382; doi:10.3389/fnut.2024.1279931)
Supplement: Supplementary file 1 [file Data_Sheet_1.docx]

***Supplementary Material***

**1 Supplementary Figures and Tables**

**1.1 Supplementary Figures**

**Supplementary Figure 1.** Screenshot of Survey Report


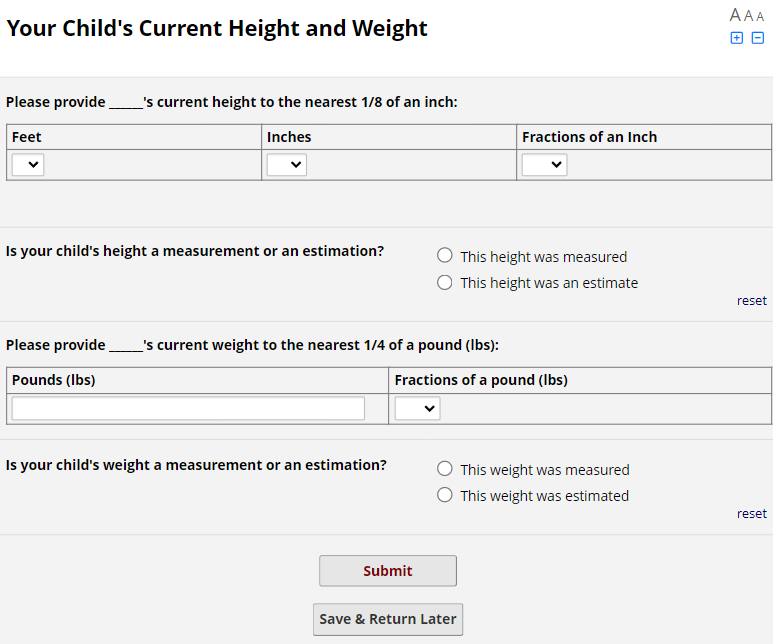


**Supplementary Figures 2A-2E.** Bland-Altman plots for parent-reported and objectively measured A) Weight (kg), B) Height (cm), C) BMI (kg/m^2^), D) BMI z-score, and E) BMI percentile among the overall subject population. The dotted line and the dashed line represent the 95% upper and lower limits of agreement (LOA), respectively. The dotted LOA is represented as +1.96 SD, the dashed LOA is represented as -1.96 SD. The central solid line represents the mean.

**2A. Weight (kg)**

**2B. Height (cm)**

**2C. BMI (kg/m^2^)**

**2D. BMI z-score**

**2E. BMI Percentile**
